# 造血干细胞移植后闭塞性细支气管炎综合征诊断与治疗中国专家共识（2022年版）

**DOI:** 10.3760/cma.j.issn.0253-2727.2022.06.001

**Published:** 2022-06

**Authors:** 中国医师协会血液科医师分会 中华医学会血液学分会

闭塞性细支气管炎综合征（bronchiolitis obliterans syndrome, BOS）是造血干细胞移植（HSCT）后晚期死亡的主要原因之一，严重限制患者的日常活动能力，导致生活质量显著降低[1]–[2]。BOS通常起病隐匿，早期无症状，但出现明显症状时肺功能已严重受损，最终可发生呼吸功能衰竭甚至死亡。由于BOS早期诊断困难，且缺乏规范化诊治的共识和标准的临床路径，制定本共识对推动HSCT技术体系的优化具有重要的临床意义[3]。为进一步规范及促进BOS的早期诊断和治疗，中国医师协会血液科医师分会和中华医学会血液学分会组织国内相关专家制定本共识。

一、BOS定义和流行病学特征

BOS是一种以HSCT后新发的持续性气流受限为特征的临床综合征。其病理特征为闭塞性细支气管炎（bronchiolitis obliterans, BO），是指细支气管损伤后的上皮炎症反应，随后的修复导致气道壁、气腔或两者兼有的肉芽组织过度增生，修复过程可以引起小气道的狭窄、扭曲（缩窄性细支气管炎）或完全闭塞（BO）。BOS可仅通过肺功能改变和影像学特征建立临床诊断，无需BO的病理学诊断，在临床工作中应用更为广泛。引起BOS的病因主要是同种异体免疫反应，也可能是由于物理化学损伤（如空气污染、毒气、吸入异物等）、感染，还有部分患者为特发性[4]。BOS可以发生于HSCT后，尤其是异基因HSCT（allo-HSCT）后。以下BOS特指HSCT后的BOS。

（一）发生率及危险因素

allo-HSCT后BOS的发生率为3％～6.5％[5]–[9]，自体HSCT后偶发[10]。BOS常发生于慢性移植物抗宿主病（cGVHD）患者，合并cGVHD时，发生率为7％～10％[9],[11]。BOS发生的最主要危险因素是进展型cGVHD，也可能与新发或静止型cGVHD、高龄、移植前气流受限、呼吸道病毒（如流感、副流感、呼吸道合胞病毒等）感染相关[12]。

（二）临床表现及预后

BOS的临床表现包括慢性干咳、劳力性呼吸困难、运动耐量下降、喘息。临床症状通常在中重度患者中出现，其中20％～30％表现为明显的气流下降。听诊呼吸音正常，或可伴有哮鸣音。40％的患者伴有低氧血症。BOS患者20％可合并胸腔漏气综合征，表现为气胸、纵隔气肿及皮下气肿[6]。另外，BOS患者易合并肺感染，若临床上发现HSCT后患者反复发生肺感染，应考虑鉴别BOS[13]。未经积极治疗BOS患者的5年生存率仅为10％～13％，随着诊疗技术的进步，其5年生存率可达40％～60％[11],[14]–[17]。目前认为与不良预后相关的因素包括：移植后早期（6～12个月）确诊BOS；确诊时第1秒用力呼气量（FEV1）低于30％预测值；确诊3个月内肺功能迅速恶化；同时合并aGVHD和其他部位cGVHD等[5],[18]–[19]。

二、BOS的辅助检查

1. 肺功能：肺功能检测是重要的辅助检查手段。BOS肺功能表现为阻塞性通气功能障碍，主要包括FEV1下降（实测值低于预测值的80％）、FEV1与用力肺活量（FVC）的比值（FEV1/FVC％）显著下降（低于70％）。FVC可在正常范围或轻度下降，肺总量（TLC）正常或增加，残气量（RV）升高（>预测值120％），残气量与肺总量比值（RV/TLC）增加（>40％）。BOS的气道阻塞病变为不可逆性，支气管舒张试验为阴性。建议：allo-HSCT后2年内，有条件的移植中心每3个月进行1次常规肺功能检查监测BOS，其后若有新出现的气流受限或其他部位新发的cGVHD表现，也应及时完善肺功能检查。

2. 高分辨率CT：高分辨率CT可发现大部分BOS患者的异常表现，但早期BOS患者也可基本正常[20]–[24]。BOS影像异常多位于双下肺和胸膜下。直接CT征象包括：外周细支气管壁增厚、小气道扩张和小叶中心性支气管结节影（细支气管扩张伴分泌物滞留）；间接CT征象包括：“空气潴留征（肺实质异常低密度衰减区，且肺体积不缩小）”、“马赛克衰减征（不同区域的肺灌注差异导致的衰减差异）”、中央型气道扩张和过度通气等。建议：高分辨率CT辅助诊断BOS应包括呼气相及吸气相扫描；主要影像表现为外周细支气管壁增厚、小气道扩张、“空气潴留征”和“马赛克衰减征”。

3. 肺组织活检：病理学检查是诊断BO的金标准，可表现为淋巴细胞性细支气管炎或缩窄性细支气管炎[25]。淋巴细胞性细支气管炎主要表现为大量淋巴细胞浸润细支气管管壁。缩窄性细支气管炎的组织学特点为细支气管黏膜下或细支气管旁纤维化，细支气管管腔向心性狭窄或完全闭塞。淋巴细胞性细支气管炎患者的肺功能受损和预后可能好于缩窄性细支气管炎患者[25]。必需注意，肺活检可能导致气胸、纵隔气肿、持续性气漏综合征甚至死亡等严重并发症，临床上应用应格外谨慎[26]。建议：在有经验和有条件的移植中心，若BOS临床诊断难以明确且患者充分知情同意，慎重作出肺穿刺活检的临床决定，避免有创操作加重病情。

三、BOS的诊断及评估

（一）诊断标准

BOS的诊断主要基于临床、影像学及肺功能检查。本共识推荐采用以下诊断标准（表1）[1],[27]–[29]。

**表1:** 闭塞性细支气管炎综合征（BOS）的诊断标准

异基因造血干细胞移植患者满足以下4条标准即可诊断：
（1）FEV1/FVC<0.7或<第5个百分位（90%置信区间下限）^a, b^。
（2）FEV1<75%预测值，在2年内下降≥10%；且在吸入β2受体激动剂（如沙丁胺醇）后FEV1增加量<200 ml，改善率<12%^b^。
（3）除外呼吸道感染，根据临床症状进行如高分辨率CT、微生物培养（鼻拭子、咽拭子、痰培养、支气管肺泡灌洗）等检查。
（4）BOS的三个相关支持证据之一：
①存在其他部位慢性GVHD表现；
②呼气相高分辨率CT显示空气潴留征、小气道壁增厚或小气道扩张；
③肺功能检查有空气滞留的证据：RV>120%预测值或RV/TLC比值>90%置信区间。

注：FEV1：第1秒用力呼气量；FVC：用力肺活量；RV：残气量；TLC：肺总量。^a^对于发育期少年儿童，肺功能指标以同年龄的肺功能百分位为准。^b^病情严重而无法进行肺功能检查、因胸廓漏气综合征的危险而不适合进行肺功能检查的患者，可以六分钟步行试验进行病情评估

（二）BOS的分度

可以根据FEV1结果将BOS分为轻度（60％～75％）、中度（40％～59％）和重度（≤39％）[28]。

（三）鉴别诊断

在BOS诊断中，排除其他病因非常重要，包括感染、隐源性机化性肺炎（COP）、晚期放疗毒性、哮喘、慢性阻塞性肺疾病等（图1）。

**图1:**
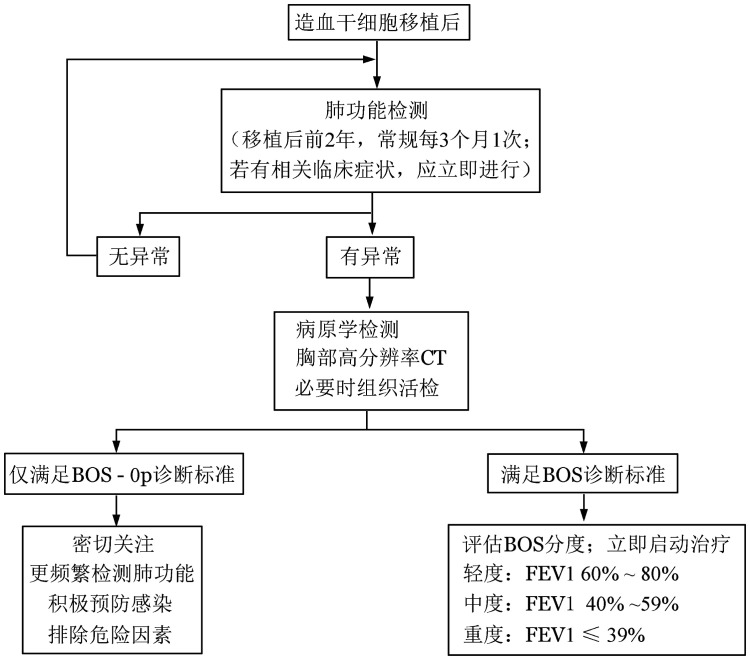
闭塞性细支气管炎综合征（BOS）的诊断及鉴别诊断流程 FEV1：第1秒用力呼气量

1. 感染：诊断BOS必须排除感染引起的细支气管炎[30]。需注意，BOS也常同时合并感染，且感染可诱发BOS加重[13]。推荐：对疑似BOS患者进行全面病原检测，包括影像学、血培养、痰培养、咽拭子培养、病毒载量测定、呼吸道病原体抗体检测、G试验、GM试验、支气管肺泡灌洗等[31]–[33]。若患者处于肺部感染状态，首先积极控制感染，再进一步明确BOS诊断。若感染反复难愈，BOS诊断困难，可在有条件的中心谨慎行活检以明确诊断。

2. 隐源性机化性肺炎（COP）：通常发生在移植后1～13个月，中位发生时间为3～4个月，症状包括发热、气短、干咳，需与HSCT后BOS鉴别。COP的肺功能通常表现为限制性通气功能障碍，主要表现为肺一氧化碳弥散（DLCO）显著下降，而FEV1/FVC比值正常；高分辨率CT常可观察到弥漫分布的实变影、支气管充气征及磨玻璃结节[34]；组织病理活检表现为远端气道及肺泡充填肉芽组织及纤维化，可伴有间质和肺泡炎症[24]。

3. 其他：迟发性肺毒性综合征在接受高剂量化疗及自体HSCT的患者中多见，可发生于移植后数月至数年，患者表现为干咳、呼吸困难和发热；但患者肺功能为限制性通气障碍，影像学提示间质纤维化改变，可以此鉴别[35]。慢性阻塞性肺疾病主要表现为干咳、喘息、劳力性呼吸困难等阻塞性通气功能障碍，与BOS患者的临床症状和肺功能改变相似，但患者往往病程长、有吸烟史、高分辨率CT无支气管壁增厚等表现。支气管哮喘为发作性伴有哮鸣音的呼气性呼吸困难，肺功能为阻塞性通气障碍；但其肺功能检查吸入β2受体激动剂（如沙丁胺醇）后FEV1的下降为可逆性。

四、BOS的治疗

BOS是一种难治性疾病，目前，其治疗在国内外均尚未形成规范化诊疗路径。本共识推荐采用联合用药方案治疗BOS，以延缓患者肺功能恶化，同时减少糖皮质激素的用量（图2）。对于肺功能恶化的难治性患者，可考虑接受肺移植术。除此之外，BOS患者应重视全身支持治疗，包括感染预防、呼吸功能锻炼、严格戒烟、营养支持等。

**图2:**
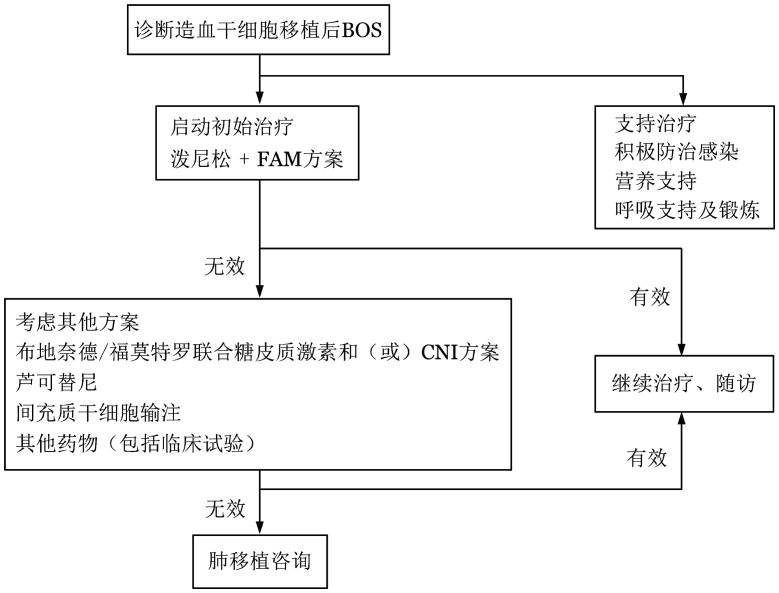
闭塞性细支气管炎综合征（BOS）的治疗流程 FAM方案：氟替卡松+阿奇霉素+孟鲁司特；CNI：钙调磷酸酶抑制剂

（一）药物治疗

1. 糖皮质激素：糖皮质激素（泼尼松）联合或不联合钙调磷酸酶抑制剂（CNI）是现今国内外cGVHD治疗的一线方案，但其在BOS中反应率欠佳，仅为20％～40％[36]–[38]。考虑到糖皮质激素的不良反应，建议：与其他药物联用以提高反应率及减少糖皮质激素用量。另外，有小规模的体内外研究显示，霉酚酸酯对于BOS的疗效可能优于环孢素A和他克莫司[39]。

2. FAM方案联合糖皮质激素：FAM方案具体用法：吸入性氟替卡松（fluticasone）440 µg每日2次；阿奇霉素（azithromycin）250 mg每周3次；孟鲁司特（montelukast）10 mg每晚1次；泼尼松1 mg·kg^−1^·d^−1^，维持2周并根据病情每周减量0.25 mg·kg^−1^·d^−1^，争取5周内减至0.25 mg·kg^−1^·d^−1^（合并其他GVHD表现而需要更高剂量患者除外）。研究证实该方案治疗3个月可明显改善36％新诊断BOS患者的肺功能，总体反应率达94％，且可显著减少糖皮质激素用量[40]–[41]。然而，阿奇霉素对BOS的肺功能改善长期疗效尚不明确[42]–[43]，故应根据患者病情决定是否以阿奇霉素作为非活动期BOS的长期用药。推荐：采用系统性糖皮质激素联合FAM方案作为BOS的初始治疗方案[44]。

3. 布地奈德/福莫特罗吸入方案：布地奈德400 µg/d+福莫特罗12 µg/d吸入每日2次。轻至重度BOS患者联合吸入布地奈德、福莫特罗并维持原有糖皮质激素或免疫抑制剂方案不变，可在治疗开始后1个月显着改善FEV1，且在6个月的研究期间维持良好疗效[45]–[46]。建议：可选择布地奈德/福莫特罗联合吸入作为糖皮质激素和（或）CNI的联合治疗方案。

4. 芦可替尼（ruxolitinib）：芦可替尼10 mg每日2次，若患者不耐受，可进行剂量调整。芦可替尼是一种JAK1/2抑制剂，可抑制促炎细胞因子和效应T细胞的生成，用于BOS的挽救性治疗可替代糖皮质激素，甚至可能改善部分患者的肺功能[47]–[51]。建议：芦可替尼可作为BOS的挽救治疗方案，尤其是合并难治性cGVHD的患者。

5. 其他：酪氨酸激酶抑制剂可通过血小板衍生的生长因子途径抑制纤维化，伊马替尼（imatinib）、尼达尼布（nintedanib）可以抑制BOS患者肺功能的恶化[50],[52]–[53]。吡非尼酮（pirfenidone）已在体内外实验被证实可显著改善肺纤维化[54]–[55]。另外，选择性Rho关联卷曲螺旋蛋白激酶2（ROCK2）抑制剂belumosudil治疗cGVHD患者BOS可获得20％～30％的缓解率，可能是潜在的BOS挽救治疗新选择[56]–[57]。建议：在有条件的临床中心，对于初始治疗无效的BOS患者，在充分知情同意情况下，可选用其挽救治疗。

（二）间充质干细胞（MSC）输注

MSC输注可调节外周淋巴细胞亚群，改善GVHD[58]，也有助于改善肺功能[59]–[60]。MSC用法：1×10^6^/kg每周1次，每4周为一个疗程。建议：在有条件的临床中心，对于BOS初始治疗无效的患者，可根据临床经验和患者病情进行MSC输注临床研究。

（三）肺移植

对于常规治疗无效且不伴有其他部位严重活动性cGVHD、无复发迹象的严重BOS患者，可以考虑进行肺移植。肺移植术后1～5年生存率可达37％～78％[61]–[63]。患者年龄较低、HSCT后2年血液病未复发、不伴有需使用免疫抑制剂治疗的其他活动性cGVHD、不伴有其他器官功能衰竭与良好的预后有关[61]。建议：对于其他治疗措施无效、原发病持续稳定、且无严重活动性cGVHD的严重BOS患者，在HSCT 1～2年后，患者充分知情同意条件下可考虑肺移植治疗。

五、BOS的疗效评估

推荐采用以下疗效评估标准：治疗反应分为：①完全缓解（CR）：治疗后FEV1/预测值比值≥80％；②部分缓解（PR）：初始FEV1/预测值<70％，治疗后FEV1/预测值比值绝对值增加10％及以上，但尚未达CR；③疾病稳定（SD）：治疗后FEV1/预测值比值绝对值增加10％～减低10％；④疾病进展（PD）：治疗后FEV1/预测值比值绝对值减低≥10％[28]。

由于BOS的疾病进展性与临床难治性，建议：评估患者维持SD状态则可认为治疗有效[28]。

六、BOS的监测

BOS患者初治时的肺功能情况（尤其是FEV1降低程度）是影响疗效和预后的重要因素[64]。因此，移植后定期肺功能筛查和BOS早期诊断十分重要。

BOS-0p阶段（BOS-0p）定义：FEV1下降>10％或中期气道流速下降>25％，但不符合BOS诊断标准，其对BOS诊断的敏感性高达85％[65]。对于这部分患者，可提高筛查肺功能的频率，监测肺功能进展；同时应尽量控制BOS发生的危险因素，包括积极预防感染、严格戒烟、防止接触物理化学毒物等。

七、BOS的预防

目前，对于BOS的预防仍处于临床试验阶段。预防性输注MSC可能对预防BOS有益[66]，该方案的安全性和有效性尚待进一步研究[67]。有研究显示阿奇霉素在HSCT后早期无助于降低BOS的发生率[43],[68]。因此，应谨慎以阿奇霉素作为BOS的预防用药，需密切监测患者的临床表现及肺功能情况。
